# *Didymosphenia geminata* in the Upper Esopus Creek: Current Status, Variability, and Controlling Factors

**DOI:** 10.1371/journal.pone.0130558

**Published:** 2015-07-06

**Authors:** Scott Daniel George, Barry Paul Baldigo

**Affiliations:** New York Water Science Center, U.S. Geological Survey, Troy, New York, United States of America; Auckland University of Technology, NEW ZEALAND

## Abstract

In May of 2009, the bloom-forming diatom *Didymosphenia geminata* was first identified in the Upper Esopus Creek, a key tributary to the New York City water-supply and a popular recreational stream. The Upper Esopus receives supplemental flows from the Shandaken Portal, an underground aqueduct delivering waters from a nearby basin. The presence of *D*. *geminata* is a concern for the local economy, water supply, and aquatic ecosystem because nuisance blooms have been linked to degraded stream condition in other regions. Here we ascertain the extent and severity of the *D*. *geminata* invasion, determine the impact of supplemental flows from the Portal on *D*. *geminata*, and identify potential factors that may limit *D*. *geminata* in the watershed. Stream temperature, discharge, and water quality were characterized at select sites and periphyton samples were collected five times at 6 to 20 study sites between 2009 and 2010 to assess standing crop, diatom community structure, and density of *D*. *geminata* and all diatoms. Density of *D*. *geminata* ranged from 0–12 cells cm^-2^ at tributary sites, 0–781 cells cm^-2 ^at sites upstream of the Portal, and 0–2,574 cells cm^-2^ at sites downstream of the Portal. Survey period and Portal (upstream or downstream) each significantly affected *D*. *geminata* cell density. In general, *D*. *geminata* was most abundant during the November 2009 and June 2010 surveys and at sites immediately downstream of the Portal. We found that *D*. *geminata* did not reach nuisance levels or strongly affect the periphyton community. Similarly, companion studies showed that local macroinvertebrate and fish communities were generally unaffected. A number of abiotic factors including variable flows and moderate levels of phosphorous and suspended sediment may limit blooms of *D*. *geminata* in this watershed.

## Introduction

The bloom-forming diatom, *Didymosphenia geminata* (Lyngbye) Schmidt, has historically been considered a wide-spread but rare species found in moderately flowing cold-water streams of North America, Europe, and Asia [1], and has more recently been introduced to New Zealand and parts of South America [2–5]. It has been termed a native invader in parts of its historical range because it has begun producing problematic blooms in some areas where it once existed in equilibrium [6–8]. Distribution patterns of *D*. *geminata* have also recently changed, resulting in greater spatial coverage and temporal persistence in streams worldwide [2]. Not only has *D*. *geminata* expanded its geographic range; evidence suggests it has also broadened its tolerance of environmental conditions. Once believed to exist only in cold, oligotrophic streams, *D*. *geminata* has now demonstrated tolerance to more nutrient-rich lotic environments [2]. In New York State, blooms of *D*. *geminata* have been confirmed in the Batten Kill (2006), East and West Branches of the Delaware River (2007 and 2008), Upper Esopus Creek (2009), Little Delaware River (2010), Neversink River (2011), Rondout Creek (2011), and others (A. Smith, New York State Department of Environmental Conservation, personal communication). Blooms of *D*. *geminata* may cover as much as 100% of stream beds with mats of extracellular mucopolysaccaride stalks that are many centimeters thick [2, 9]. The production of stalk material traps algae, macroinvertebrates, and detritus, and extensive blooms of *D*. *geminata* can severely alter benthic habitat, river hydraulics, and the condition of lotic freshwater ecosystems [9–12]. Nuisance blooms can also negatively impact recreational opportunities and local economies [13].

Major progress has been made over the past ten years towards understanding the factors that induce *D*. *geminata* to produce nuisance blooms. Blooms are caused primarily by the extensive production of stalk material and may not be associated with high rates of cell division [14, 15]. It is now believed that *D*. *geminata* produces extensive stalk material when it is phosphorous-limited, which may be a strategy to expose cells to the water column for greater acquisition of phosphorous [16–18]. Specifically, phosphatase activity in the stalks and nutrient cycling within the resulting mats may provide *D*. *geminata* with a competitive advantage over other diatoms in low-nutrient environments [19–21]. When *D*. *geminata* is not phosphorous-limited, it exhibits faster rates of cell division and may exist at comparatively higher cell densities for short periods of time [15]. Under these conditions, extensive stalk production is less common [16] and *D*. *geminata* may exist in a non-nuisance capacity. More generally, blooms of *D*. *geminata* often occur under conditions of low nutrients, high light, low temperature, and infrequent hydrologic disturbances [9, 18]. The frequency of high flow events, particularly those that mobilize the streambed, is considered the best hydrologic predictor of *D*. *geminata* biomass [2, 18]. Bed-mobilizing events scour away existing periphyton biomass and effectively reset the periphyton successional process. Because *D*. *geminata* may be a late successional species [22], frequent high-flow events can limit cell density and stalk biomass. Thus, it is clear why *D*. *geminata* thrives, and sometimes reaches nuisance levels, in streams below impoundments which moderate flows and water temperature [2, 23].

In May 2009, *D*. *geminata* was first identified in the Upper Esopus Creek, a key tributary to the New York City water-supply and a popular recreational stream. Although it is unknown if *D*. *geminata* is native to the Upper Esopus, subfossil records indicate that it was historically present on Long Island [24] and at the mouth of the Delaware River in New Jersey [25], both of which drain this region [8]. Regardless, water quality and aquatic biota are extensively monitored in the Upper Esopus and it is unlikely that significant blooms occurred prior to 2009. The identification of *D*. *geminata* is concerning because nuisance blooms could threaten aquatic food webs, recreation (fishing and tubing), and therefore the regional economies that depend on the Upper Esopus and other Catskill Mountain Rivers. Although recent publications have helped better define the effects of watershed and water quality parameters on *D*. *geminata*, the basic ecological knowledge necessary to design management strategies that might control or mitigate nuisance blooms is still limited [2, 9, 10, 26]. The primary objectives of this study are to: 1) ascertain the current extent and severity of the *D*. *geminata* invasion, 2) determine the impact of supplemental flows from an inter-basin aqueduct on *D*. *geminata*, and 3) identify potential limiting factors for *D*. *geminata* in the Upper Esopus Creek watershed.

## Methods

### Ethics and data availability statement

Study sites were distributed across public and private property and landowner permission was obtained prior to sampling at privately owned properties. Permits were not required and no protected species were sampled during this project. All relevant data are included herein and thus are publically available.

### Study scope and area

**Fig 1:**
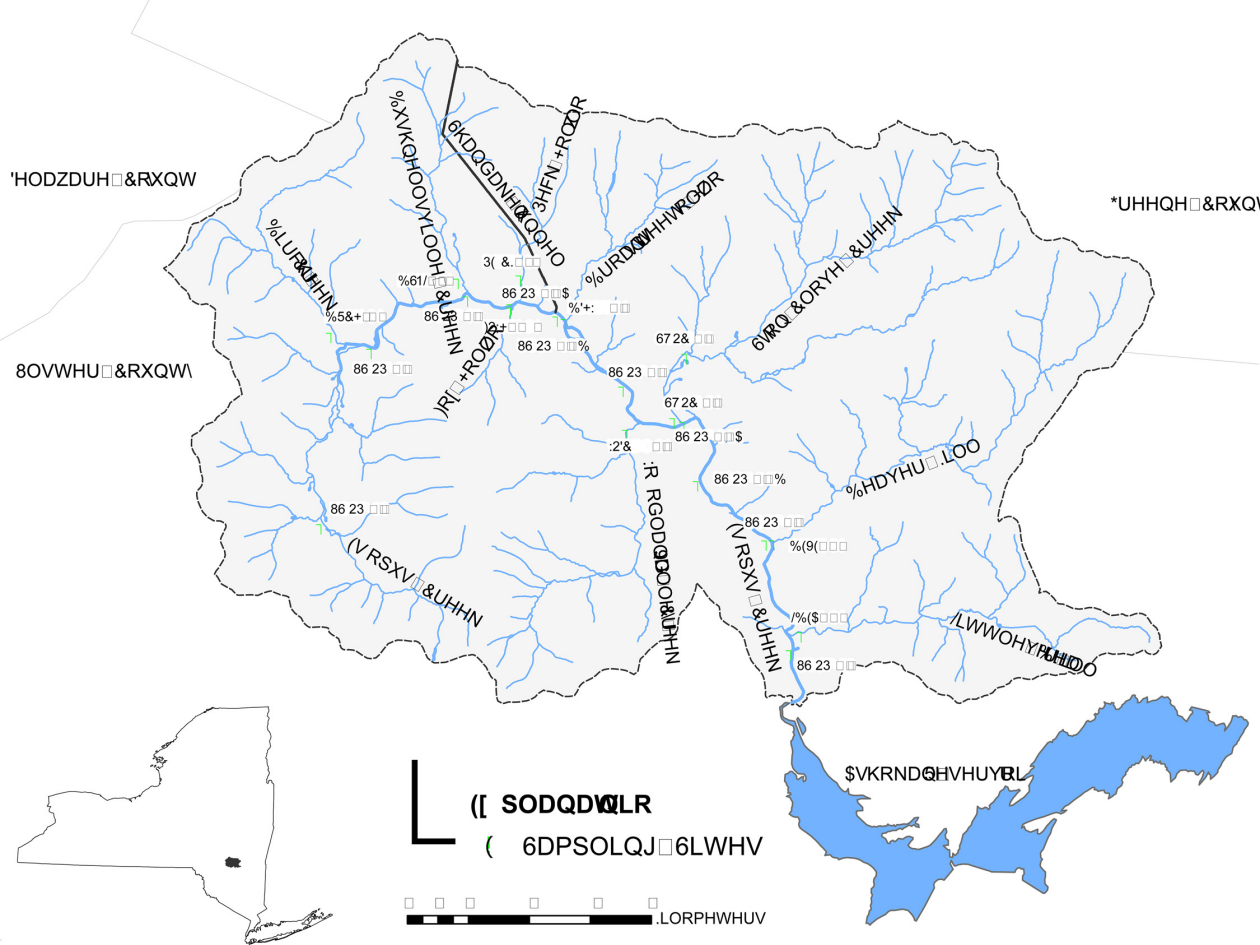
Locations of periphyton sampling sites in the Upper Esopus Creek and tributaries, 2009–2010.

The Upper Esopus Creek is located in the south central Catskill Mountain Region of southeastern New York (Fig 1). The Creek follows a 41.8 km semi-circular course from its headwaters at Winnisook Lake, around Panther Mountain, to its impoundment downstream of Boiceville, where it forms the Ashokan Reservoir. The watershed area of the Upper Esopus Creek is 497.3 km^2^ and drains some of the most rugged and mountainous terrain in the Catskills. Forested land comprises over 95% of the watershed and its surficial geology features lacustrine clay deposits that contribute suspended sediment to the system [27]. Turbidity and other potential water quality impairments are a major concern in this watershed because the Ashokan Reservoir provides close to 40% of New York City’s drinking water [28]. Nine major tributaries (Table 1) deliver waters to the Upper Esopus in addition to the Shandaken Portal, the terminus of an inter-basin aqueduct which diverts water from Schoharie Reservoir to its confluence with the Upper Esopus in Shandaken. Discharge from the Portal can increase natural flows on the Upper Esopus by a factor of two or greater and the supplemental flow usually has a moderating effect on ambient stream temperature (cooler in the summer, warmer in the winter) [29].

**Table 1:** Stream name, site code, coordinates, drainage area (DA), and elevation for periphyton surveys conducted on the Upper Esopus Creek and tributaries, 2009–2010. Asterisks denote seasonal sites that were sampled during all five surveys.

Stream name	Site code	Latitude	Longitude	DA (km^2^)	Elevation (m)
Tributary sites					
Fox Hollow	FOXH-01	42.116111	-74.380556	10.3	309.4
Peck Hollow	PECK-01	42.125556	-74.376389	12.3	350.5
Broadstreet Hollow	BDHW-01	42.112556	-74.358694	23.7	295.8
Bushnellsville Creek	BSNL-01	42.124722	-74.401139	29.5	336.4
Birch Creek	BRCH-04	42.108979	-74.451818	32.4	377.4
Little Beaver Kill	LBEA-01	42.019536	-74.266258	42.7	204.7
Woodland Valley Creek	WODC-01	42.079722	-74.334583	53.4	267.6
Beaver Kill	BEVE-01	42.046758	-74.276814	64.7	213.5
Stony Clove Creek at Chichester	STOC-00	42.102028	-74.310889	80.0	291.6
Stony Clove Creek at Phoenicia	STOC-01	42.083056	-74.315833	83.9	245.2
Main stem sites					
Esopus Creek at Oliverea	USOP-00	42.052500	-74.456222	30.3	454.5
Esopus Creek at Big Indian	USOP-02*	42.104167	-74.435833	111.9	354.9
Esopus Creek at Shandaken	USOP-03*	42.119444	-74.397500	152.0	316.8
Esopus Creek at Allaben	USOP-03A*	42.117034	-74.380149	165.0	304.6
Esopus Creek downstream of Portal	USOP-03B*	42.113333	-74.361889	181.0	287.2
Esopus Creek upstream of Phoenicia	USOP-04	42.092500	-74.335972	215.7	268.0
Esopus Creek at Phoenicia	USOP-04A*	42.081944	-74.312028	357.4	237.5
Esopus Creek downstream of Phoenicia	USOP-04B	42.063611	-74.306389	365.2	225.3
Esopus Creek at Mt. Tremper	USOP-05	42.046889	-74.280000	373.0	207.4
Esopus Creek at Boiceville	USOP-06*	42.014259	-74.270425	497.3	188.8

Periphyton samples were collected from 20 study sites on two occasions and from six of these sites on three other occasions for a total of five surveys between 2009 and 2010. During August 2009 and August 2010, periphyton samples were collected from all 20 study sites across the watershed (Table 1). Ten sites were located on the Upper Esopus, including four upstream and six downstream of the Shandaken Portal. Nine other sites were located on tributaries near their confluences with the Upper Esopus, and the last site was approximately 3 km upstream in the largest tributary, Stony Clove Creek (Fig 1). Periphyton samples were also collected from six main stem sites (three upstream and three downstream of the Portal) during November 2009, April 2010, and June 2010 to assess seasonal variation (herein termed seasonal sites).

### Periphyton sampling

Although this study did not quantitatively define a bloom, most field studies have found that blooms are positively correlated with cell density [6, 19], and thus cell density of *D*. *geminata* and periphyton standing crop are used to estimate the spatiotemporal variability of *D*. *geminata*. Periphyton samples were collected using methods described in the U.S. Environmental Protection Agency periphyton protocol for single habitat sampling [30]. Periphyton was sampled from riffles because assemblages within a single habitat type are more homogeneous than those from across multiple habitats, and therefore are more sensitive to subtle differences in water quality [30].

Quantitative periphyton samples were collected to determine standing crop of periphyton using chlorophyll *a* (chl *a*) and ash-free dry mass (AFDM) and to identify attached diatoms as follows. Three replicate samples were collected at each site: one near the left bank, one near the right bank, and one from the center of the channel. For each replicate, the scrapings were composited from a delineated area of the surface of three rocks classified as boulder or large cobble using the Wentworth Scale [31]. The volume of the slurry was measured and subsamples were taken for determination of chl *a*, AFDM, and diatom identification. For chl *a* and AFDM, the sample was mixed thoroughly with an electric mixer and a 5-mL subsample was vacuumed through a glass fiber filter. Each filter was placed in a petri dish, covered with foil, and kept on ice until it could be frozen. For diatom identification, the sample was mixed with an electric mixer and a 20-mL subsample was placed in a glass vial and preserved with 5 mL of formalin. The scraped area of each rock was outlined in chalk, overlain with a wire screen of known mesh size, and photographed. Total area of each rock scrape was calculated from these photographs using digital image analysis software [32].

Chlorophyll *a* concentrations were determined using standard fluorometric methods with a correction for pheophytin *a* [33]. Filters from each sample were extracted in acetone and centrifuged, and fluorescence was read before and after the addition of hydrochloric acid. Hold time for chl *a* samples ranged from roughly 4–13 months depending on sample date. The total chl *a* concentration was expressed as μg cm^-2^. Ash-free dry mass was calculated as the difference between the dried weight and ashed weight of filters and expressed as mg cm^-2^. Filters were oven dried at 100°C for 24 h, weighed, ashed at 500°C for 2 h, and reweighed to determine AFDM [34]. The AFDM for three sites (USOP-03B, USOP-04A, and USOP-06) sampled in June 2010 could not be determined because cellulose filters were used. These filters could not be ashed and could have affected chl *a* measurements at these sites as well.

Samples for diatom identification were shipped to a contract laboratory (Rhithron Associates, Inc., Missoula, Montana) and identified to lowest possible taxon (generally species). Permanent diatom slides were prepared from acid-washed subsamples from each replicate at each site. A transect was scribed on each slide and the first 600 valves (300 cells) along the transect were identified. Unfortunately, upon reviewing the data it became apparent that this method did not sufficiently document *D*. *geminata* cell density. In some samples, *D*. *geminata* was not among the first 600 diatoms identified, yet a brief visual scan of the slide clearly showed it was abundant. It has been demonstrated that such fixed count methods are biased towards smaller diatoms [2, 35]. Because *D*. *geminata* cells are very large relative to other diatoms, it was hypothesized that this methodology was insufficient to meet the study objectives. Consequently, all slides were rescanned entirely (no transects used) and all *D*. *geminata* cells were counted. The density of the entire diatom community was calculated using the ratio of area needed to count 300 cells along a transect = area of the entire slide/total number of cells on the slide. The laboratory pipetted approximately 0.6 mL onto each slide, which enabled the density of both *D*. *geminata* and the entire diatom community to be expressed in terms of cells per square centimeter of rock surface area.

### Statistical analysis

Spearman correlations were used to assess relationships between *D*. *geminata* cell density, total diatom cell density, measures of standing crop, and basic hydrologic variables (mean discharge, discharge coefficient of variation (CV), and mean temperature of the 30 days preceding each survey) which were collected by or modeled from USGS stream gages (Table 2). A general linear mixed effects model was used to assess spatial and temporal differences in log(x+1)-transformed *D*. *geminata* cell density data with survey period, Portal (upstream or downstream), and the interaction term period*Portal as fixed factors and site (nested within Portal) as a random factor to account for repeated sampling of sites over time. Pairwise comparisons of significant effects were conducted with Tukey’s HSD test. The above analyses were performed on the mean values of the three rock scrape replicates, only considered the six seasonal sites (Table 1), and were conducted using Minitab v17.1 software.

**Table 2:** Mean temperature, mean daily discharge, and discharge coefficient of variation (CV) for the 30 days preceding each survey at seasonal sites.

Location	Period	Mean temperature (°C)	Mean discharge (m^3^ s^-1^)	CV discharge^1^
USOP-02	Aug 2009	15.5	4.1	1.26
USOP-03	Aug 2009	15.5	5.5	1.26
USOP-03A	Aug 2009	16.1	6.0	1.26
USOP-03B	Aug 2009	15.3	10.5	0.70
USOP-04A	Aug 2009	16.4	18.7	0.99
USOP-06	Aug 2009	18.0	24.4	1.06
USOP-02	Nov 2009	10.2	2.2	0.99
USOP-03	Nov 2009	10.5	3.0	0.99
USOP-03A	Nov 2009	9.1	3.2	0.99
USOP-03B	Nov 2009	11.8	9.0	0.25
USOP-04A	Nov 2009	10.3	14.3	0.62
USOP-06	Nov 2009	11.1	17.7	0.73
USOP-02	Apr 2010	5.4	18.4	0.71
USOP-03	Apr 2010	5.9	25.0	0.71
USOP-03A	Apr 2010	6.0	27.2	0.71
USOP-03B	Apr 2010	6.0	29.7	0.73
USOP-04A	Apr 2010	6.6	54.1	0.84
USOP-06	Apr 2010	6.9	75.2	0.84
USOP-02	Jun 2010	14.5	1.4	0.34
USOP-03	Jun 2010	14.6	1.9	0.34
USOP-03A	Jun 2010	14.8	2.0	0.34
USOP-03B	Jun 2010	12.3	11.8	0.53
USOP-04A	Jun 2010	13.3	13.4	0.45
USOP-06	Jun 2010	15.2	14.8	0.42
USOP-02	Aug 2010	19.6	0.4	0.34
USOP-03	Aug 2010	19.0	0.5	0.34
USOP-03A	Aug 2010	19.7	0.5	0.34
USOP-03B	Aug 2010	20.5	5.5	0.12
USOP-04A	Aug 2010	20.7	6.0	0.11
USOP-06	Aug 2010	21.9	6.4	0.11

^1^ Discharge at USOP-02 and USOP-03 were modeled from USGS stream gage data at USOP-03A using a drainage area correction and therefore have the same coefficient of variation.

Results of the diatom identifications were used to assess community structure using multivariate techniques with Primer-E v6 software with PERMANOVA+ [36–38]. The replicates for each sample were combined, fourth-root transformed, and used to form a resemblance matrix of Bray-Curtis similarities comparing all samples. Samples were plotted in “species-space” on a non-metric multidimensional scaling (MDS) ordination [39, 40] according to the non-parametric ranks of their Bray-Curtis similarities [36]. A two-way crossed Analysis of Similarities (ANOSIM) test was applied to the resemblance matrix to test for significant effects of period and density class of *D*. *geminata* (no detection, 0–100 cells cm^-2^, >100 cells cm^-2^) on the diatom community. The homogeneity of multivariate dispersion within groups was assessed using PERMDISP [37]. Although the ANOSIM test produces *P*-values, the value of the R statistic is considered more important for assessing differences between groups [36]. An R value of less than 0.25 indicates barely separable groups, whereas an R value of greater than 0.5 indicates separate but overlapping groups, and values greater than 0.75 indicate well separated groups [41]. It was hypothesized that the composition of the diatom community would be altered at sites where *D*. *geminata* was present or abundant and that these sites would group separately in the ordination.

## Results

Density of *D*. *geminata* and total diatoms, percentage *D*. *geminata*, chl *a*, and AFDM values are presented in Table 3 as means and standard deviations of the three replicates collected at each site. Density of *D*. *geminata* ranged from 0 cells cm^-2^ (observed frequently) to 2,574 cells cm^-2^ (observed at USOP-03B in November 2009). Total diatom density ranged from 19,085 cells cm^-2^ at USOP-04A during April 2010 to 419,956 cells cm^-2^ at USOP-03A during November 2009. The lowest chl *a* (0.82 μg cm^-2^) and AFDM (0.36 mg cm^-2^) concentrations were observed at USOP-00 during August 2009 and the highest chl *a* (20.77 μg cm^-2^) and AFDM (5.98 mg cm^-2^) concentrations occurred at USOP-03B during November 2009. Density of *D*. *geminata* was significantly correlated with total diatom density (*r* = 0.42, *P* = 0.022) and inversely correlated with CV of discharge (*r =* -0.52, *P* = 0.003) but was not significantly correlated with chl *a* (*r* = -0.05, *P* = 0.795), AFDM (*r* = 0.05, *P* = 0.810), mean discharge (*r* = -0.10, *P* = 0.590), or mean temperature (*r* = 0.24, *P* = 0.202). Total diatom density was significantly correlated with chl *a* (*r* = 0.43, *P* = 0.018) and AFDM (*r* = 0.63, *P* = 0.000) and standing crop measures (AFDM and chl *a*) were significantly correlated (*r* = 0.90, *P* = 0.000).

**Table 3:** Summary information for *D*. *geminata* density, total diatom density, percent *D*. *geminata*, chl *a*, and AFDM by site and survey period.

Site	Date (mm/dd/yyyy)	*D*. *geminata* density (cells cm^-2^)		Total diatom density (cells cm^-2^)		% *D*. *geminata* (relative abundance)		Chl *a* (μg cm^-2^)		AFDM (mg cm^-2^)	
		Mean	SD	Mean	SD	Mean	SD	Mean	SD	Mean	SD
August 2009											
USOP-00	8/26/2009	-	-	32770	23098	-	-	0.82	0.23	0.36	0.12
USOP-02	8/26/2009	-	-	24040	13657	-	-	2.50	1.77	0.80	0.39
USOP-03	8/26/2009	-	-	122589	140194	-	-	2.43	1.62	1.08	0.81
USOP-03A	8/26/2009	-	-	144622	100559	-	-	4.53	3.04	1.75	1.21
USOP-03B	8/27/2009	32.7	44.3	65687	38471	0.040	0.038	1.97	0.35	0.78	0.13
USOP-04	8/27/2009	0.7	0.9	53529	24070	0.002	0.002	1.84	1.01	0.67	0.26
USOP-04A	8/27/2009	9.5	15.6	81673	65475	0.007	0.009	1.80	0.45	0.47	0.06
USOP-04B	8/27/2009	10.9	4.7	154168	93078	0.012	0.012	4.22	1.12	0.96	0.33
USOP-05	8/27/2009	247.4	405.9	201486	224062	0.062	0.082	3.81	1.84	1.62	0.33
USOP-06	8/27/2009	6.2	3.3	146402	46029	0.004	0.001	3.95	0.18	1.85	0.78
FOXH-01	8/25/2009	-	-	163394	34572	-	-	6.15	3.55	1.33	0.71
PECK-01	8/25/2009	-	-	156736	40769	-	-	3.36	0.43	0.73	0.09
BDHW-01	8/25/2009	-	-	242099	55396	-	-	9.63	1.14	1.37	0.13
BSNL-01	8/25/2009	-	-	126442	52466	-	-	3.16	1.67	0.77	0.25
BRCH-04	8/25/2009	-	-	101966	5187	-	-	5.93	2.77	1.40	0.63
LBEA-01	8/26/2009	-	-	118870	34599	-	-	6.48	2.86	0.92	0.34
WODC-01	8/26/2009	0.3	0.6	277116	118441	<0.001	<0.001	4.87	0.91	0.89	0.17
BEVE-01	8/26/2009	-	-	251412	50248	-	-	4.06	1.97	1.05	0.36
STOC-00	8/26/2009	12.2	3.1	82480	32260	0.016	0.005	2.67	0.16	0.62	0.08
STOC-01	8/26/2009	2.0	1.0	257325	166689	0.001	0.001	7.45	3.49	1.18	0.31
November 2009											
USOP-02	11/3/2009	-	-	367107	263146	-	-	11.27	5.80	2.59	1.28
USOP-03	11/3/2009	-	-	362455	57761	-	-	12.40	1.94	2.33	0.16
USOP-03A	11/3/2009	24.1	33.5	419956	189425	0.004	0.005	13.10	3.48	2.95	0.95
USOP-03B	11/3/2009	2573.5	1259.9	212550	137401	1.334	0.665	20.77	6.66	5.98	2.54
USOP-04A	11/3/2009	1409.5	1506.4	310396	149016	0.434	0.316	11.15	3.43	3.49	1.01
USOP-06	11/3/2009	72.5	121.2	231699	125699	0.020	0.032	6.59	1.49	1.78	0.24
April 2010											
USOP-02	4/13/2010	-	-	133040	183676	-	-	15.43	7.67	2.25	1.19
USOP-03	4/13/2010	-	-	39451	43987	-	-	14.82	4.74	1.85	0.47
USOP-03A	4/13/2010	-	-	66075	14849	-	-	7.91	2.52	1.83	0.23
USOP-03B	4/13/2010	-	-	72088	52330	-	-	13.56	2.07	2.05	0.28
USOP-04A	4/13/2010	-	-	19085	6844	-	-	1.82	1.32	0.51	0.28
USOP-06	4/13/2010	-	-	83589	66375	-	-	2.94	1.14	0.67	0.24
June 2010											
USOP-02	6/17/2010	0.7	1.2	55372	41474	0.001	0.001	1.80	0.98	0.61	0.21
USOP-03	6/17/2010	116.9	143.4	112084	20981	0.125	0.169	3.08	0.94	0.67	0.13
USOP-03A	6/17/2010	781.1	525.8	135424	73531	0.566	0.364	3.35	0.48	1.13	0.24
USOP-03B	6/17/2010	470.9	573.7	185534	27162	0.291	0.384	4.54	1.23	-	-
USOP-04A	6/17/2010	937.4	465.8	226939	69308	0.441	0.255	7.34	1.90	-	-
USOP-06	6/17/2010	1421.8	506.5	167346	23157	0.849	0.260	3.86	1.80	-	-
August 2010											
USOP-00	8/16/2010	-	-	175548	49161	-	-	6.75	1.30	1.39	0.29
USOP-02	8/17/2010	-	-	51252	5171	-	-	4.86	1.34	1.35	0.64
USOP-03	8/17/2010	2.7	1.6	173665	35660	0.002	0.001	4.41	0.61	1.47	0.35
USOP-03A	8/17/2010	7.1	11.3	152355	65050	0.003	0.005	6.85	5.32	2.17	0.76
USOP-03B	8/17/2010	108.9	126.8	81326	25222	0.136	0.173	1.40	0.36	0.99	0.21
USOP-04	8/18/2010	1.9	1.0	53201	19178	0.004	0.003	3.39	0.50	0.92	0.08
USOP-04A	8/17/2010	12.7	12.6	76258	6957	0.016	0.015	5.51	2.13	1.80	0.96
USOP-04B	8/17/2010	6.3	5.3	66239	31310	0.013	0.012	5.22	1.31	1.43	0.08
USOP-05	8/17/2010	17.0	13.9	91150	38411	0.022	0.021	5.48	1.57	1.37	0.37
USOP-06	8/17/2010	7.0	1.3	29979	13455	0.025	0.006	5.83	2.47	1.20	0.17
FOXH-01	8/16/2010	-	-	131476	48224	-	-	5.84	2.95	1.22	0.50
PECK-01	8/16/2010	-	-	131946	39447	-	-	8.82	2.89	1.09	0.44
BDHW-01	8/16/2010	-	-	83931	19967	-	-	7.05	0.89	1.12	0.10
BSNL-01	8/16/2010	0.5	0.4	110333	45591	0.001	0.001	6.86	2.45	1.09	0.19
BRCH-04	8/16/2010	-	-	113790	50143	-	-	8.42	1.41	1.68	0.11
LBEA-01	8/18/2010	-	-	203032	76952	-	-	6.99	4.00	1.09	0.12
WODC-01	8/16/2010	-	-	76183	13011	-	-	2.98	0.48	0.95	0.16
BEVE-01	8/18/2010	-	-	133482	6141	-	-	5.30	2.96	2.18	0.93
STOC-00	8/18/2010	0.1	0.3	63253	3476	<0.001	<0.001	3.15	0.49	0.91	0.07
STOC-01	8/16/2010	0.3	0.5	46803	20731	0.001	0.001	6.31	1.15	1.40	0.50

### Temporal variation

**Fig 2:**
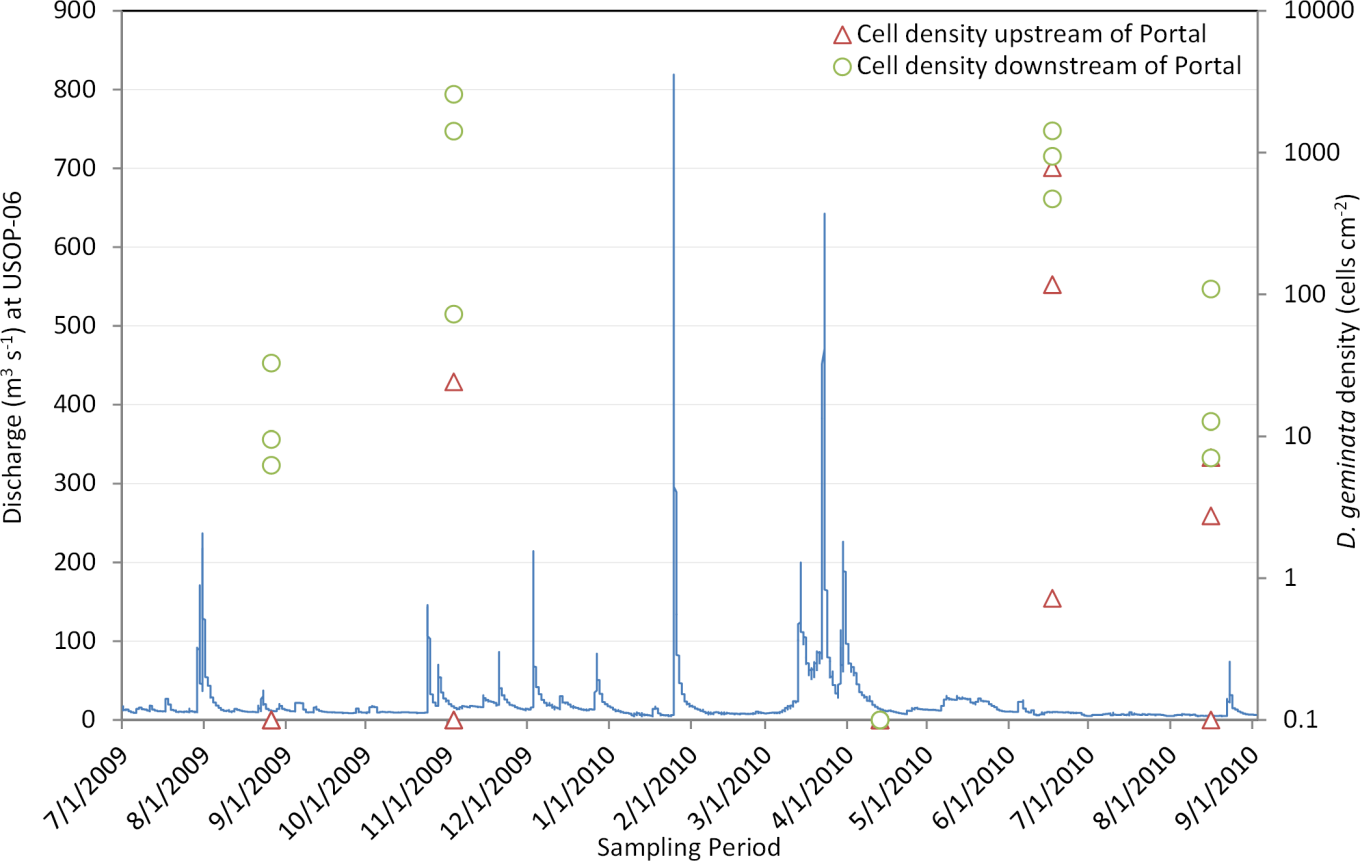
Continuous (15-minute) discharge at USOP-06 and cell density of *D*. *geminata* from seasonal sites upstream of the Shandaken Portal (red triangles) and downstream of the Shandaken Portal (green circles) during the five surveys conducted between 8/1/2009 and 9/1/2010 [all zero values (no detection) were replaced with 0.1 to facilitate plotting in log space].

**Fig 3:**
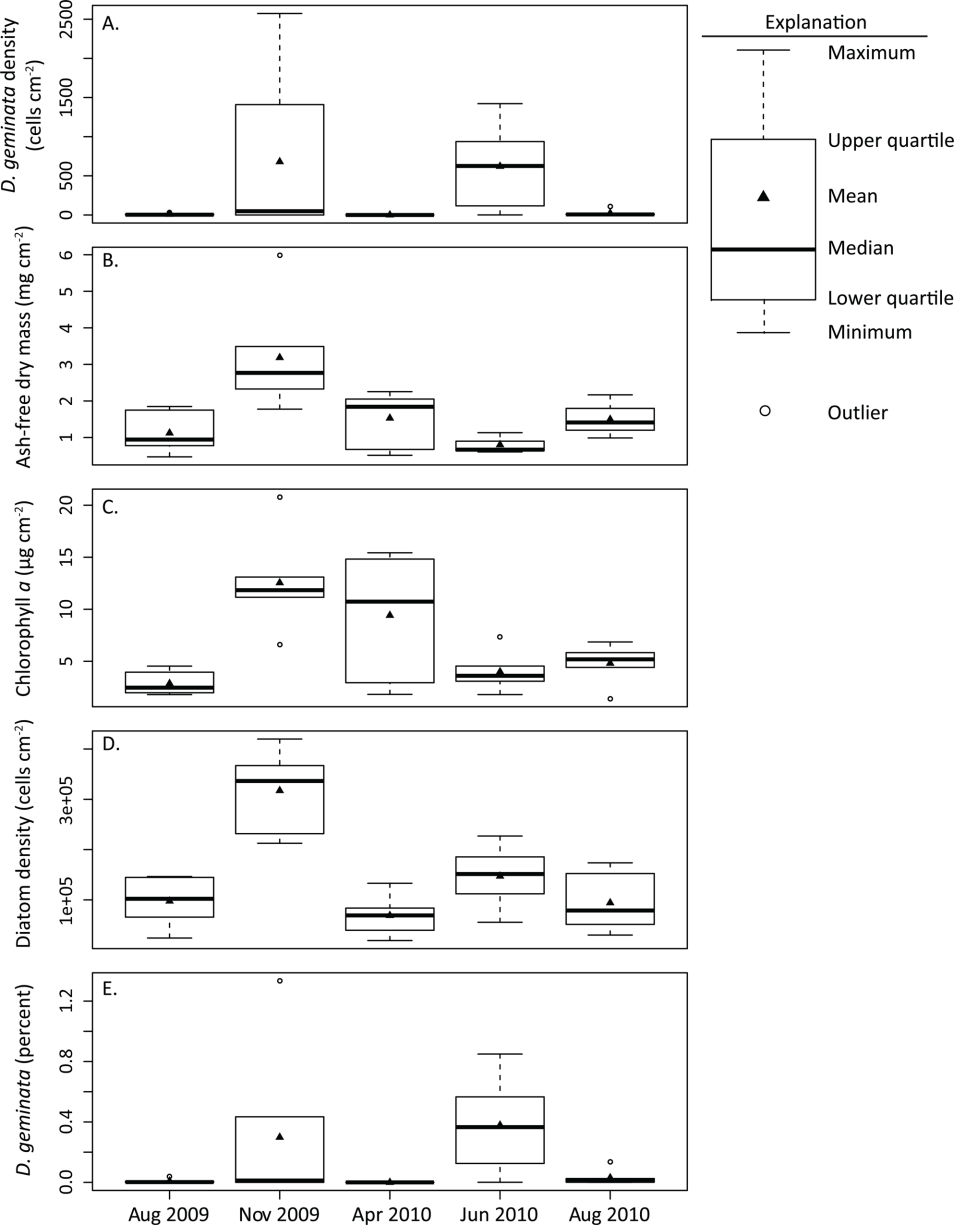
Box and whisker plots summarizing temporal trends in (A) *D*. *geminata* density, (B) ash-free dry mass (AFDM), (C) chlorophyll *a* (chl *a*), (D) total diatom density, and (E) percent *D*. *geminata* for all surveys conducted at seasonal sites, 2009–2010. Median value is indicated by the black center line, mean value is indicated by the black triangle, and the bottom and top of the box indicate the lower and upper quartiles, respectively. Whiskers represent the minimum and maximum values and hollow circles represent outliers.

The density of *D*. *geminata* was high and variable at many sites during the November 2009 and June 2010 surveys while density was consistently low during the August 2009 and August 2010 surveys (Fig 2). No *D*. *geminata* cells were collected at any of the six sites during the April 2010 survey. The mixed model confirmed that period had a significant effect (*P* = 0.000) on the density of *D*. *geminata* (Table 4) and pairwise comparisons indicated the following grouping: June 2010: A, November 2009: AB, August 2010: BC, August 2009: C, April 2010: C (periods that do not share a letter are significantly different).Temporal changes in density of *D*. *geminata* were generally consistent with total diatom density and standing crop for most surveys. *D*. *geminata* cell density (Fig 3A), AFDM (Fig 3B), chl *a* (Fig 3C), and total diatom density (Fig 3D) were concurrently high during November 2009 and low during August 2009 and 2010. This relationship was not maintained in the April 2010 survey as no *D*. *geminata* cells were detected and total diatom density was low, yet standing crop measures were relatively high. Density of *D*. *geminata* and total diatoms was high in June 2010, but AFDM and chl *a* concentrations were relatively low during this survey.

**Table 4:** Results of mixed model analysis of log(x+1)-transformed *D*. *geminata* cell density with Portal, period, and period*Portal as fixed factors and site (nested within Portal) as a random factor.

Factor	Degrees of Freedom	F-value	*P*-value
Portal	1	11.22	0.029
Period	4	17.43	0.000
Period*Portal	4	3.68	0.026

The relative abundance of *D*. *geminata* ranged from 0–1.3% (Table 3) of the entire diatom community and generally increased during the same periods when total diatom density increased (Fig 3E). Increases in the percentage of *D*. *geminata* coincided with increases in AFDM and chl *a* during the November 2009 survey but not during the June 2010 survey. During the latter period, mean *D*. *geminata* density (621 cells cm^-2^) and relative abundance (0.4%) were among the highest observed during the study, yet mean chl *a* and the limited AFDM values were at moderate to low values (4.00 μg cm^-2^ and 0.80 mg cm^-2^, respectively).

**Fig 4:**
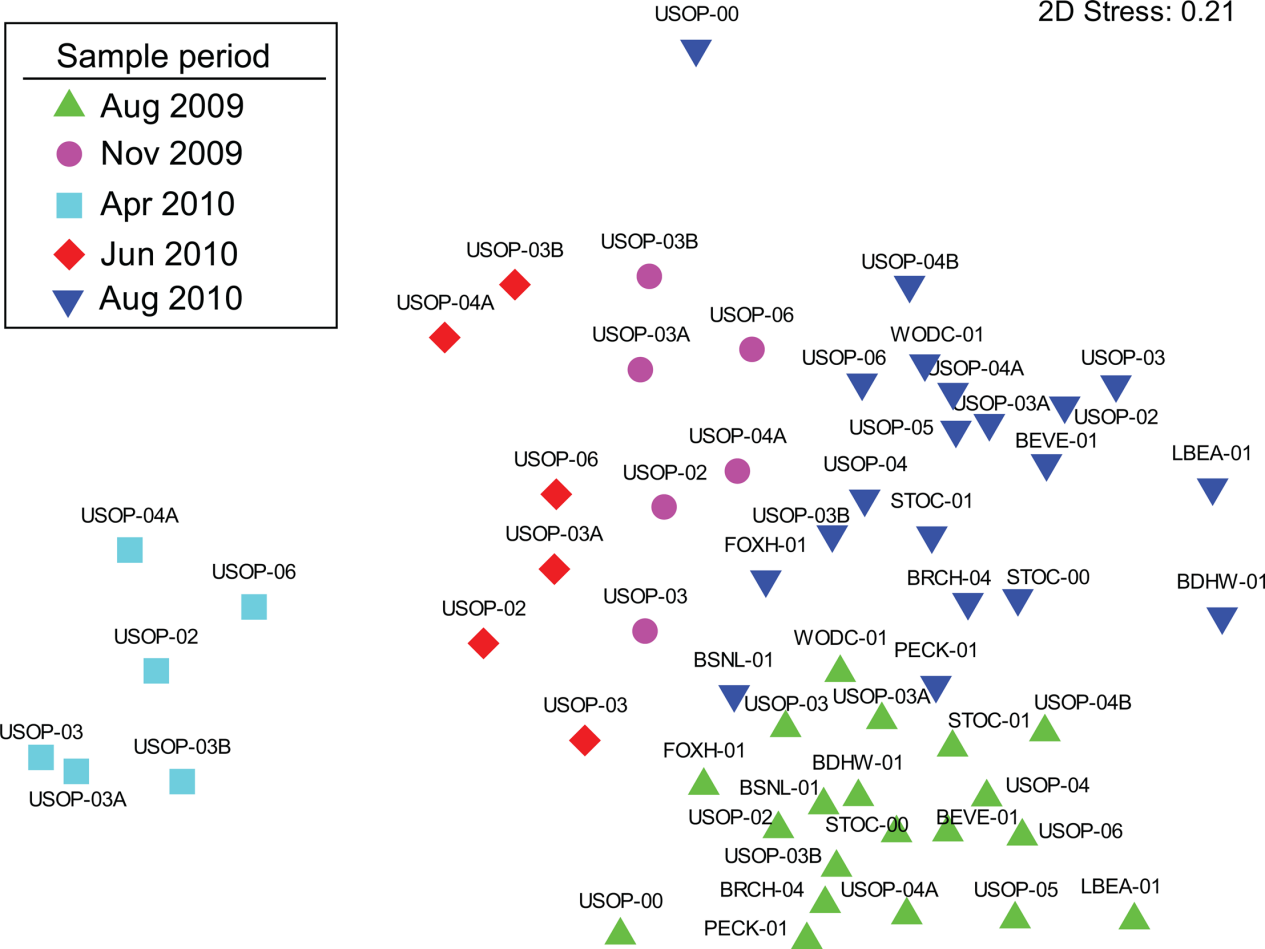
MDS ordination based on fourth-root transformed diatom abundance data for each of the five surveys as indicated by colored symbols.

Diatom community structure was strongly influenced by survey period but not by the cell density of *D*. *geminata*. Assemblages from each period clustered tightly in the MDS and April 2010 samples were most strongly isolated (Fig 4). A two-way ANOSIM test confirmed that differences between survey period were highly significant (Global R: 0.764, *P* = 0.001) and all pairwise comparisons between periods were significant (*P*<0.05). The density class of *D*. *geminata* (no detection, 0–100 cells cm^-2^, >100 cells cm^-2^) did not significantly affect the composition of the diatom community (Global R: 0.106, *P* = 0.053). PERMDISP indicated that multivariate dispersion (community homogeneity) differed significantly between periods (*P* = 0.002) and density class of *D*. *geminata* (*P* = 0.014). Pairwise comparisons indicated that diatom assemblages from the >100 cells cm^-2^ class were significantly more homogenous than the no detection class (*P* = 0.023) but did not differ from the 0–100 cells cm^-2^ class (*P* = 0.311). Sites where *D*. *geminata* was present in high densities, however, did not consistently separate in the ordination from nearby sites where it was not detected. For example, during the November survey, diatom assemblages at USOP-02 and USOP-04A had a high degree of similarity and were located close to one another in the ordination yet *D*. *geminata* was not detected at USOP-02 and was present at a density of 1,410 cells cm^-2^ at USOP-04A.

### Spatial variation

Spatial differences in the density of *D*. *geminata* were clearly evident during the study. Cell densities at seasonal sites were generally lowest at upstream sites, peaked abruptly at USOP-03B, and then declined gradually at sites further downstream. The highest mean density of *D*. *geminata* (637 cells cm^-2^) was observed at USOP-03B which is located immediately downstream from the confluence of the Shandaken Portal with the Upper Esopus. Portal was a significant (*P* = 0.029) factor in the mixed model and indicated that sites downstream of the Portal had greater densities of *D*. *geminata* than sites upstream of the Portal (Table 4). The interaction term period*Portal was also significant (*P* = 0.026) which suggests the effect of the Portal on *D*. *geminata* cell density differed by period.

**Fig 5:**
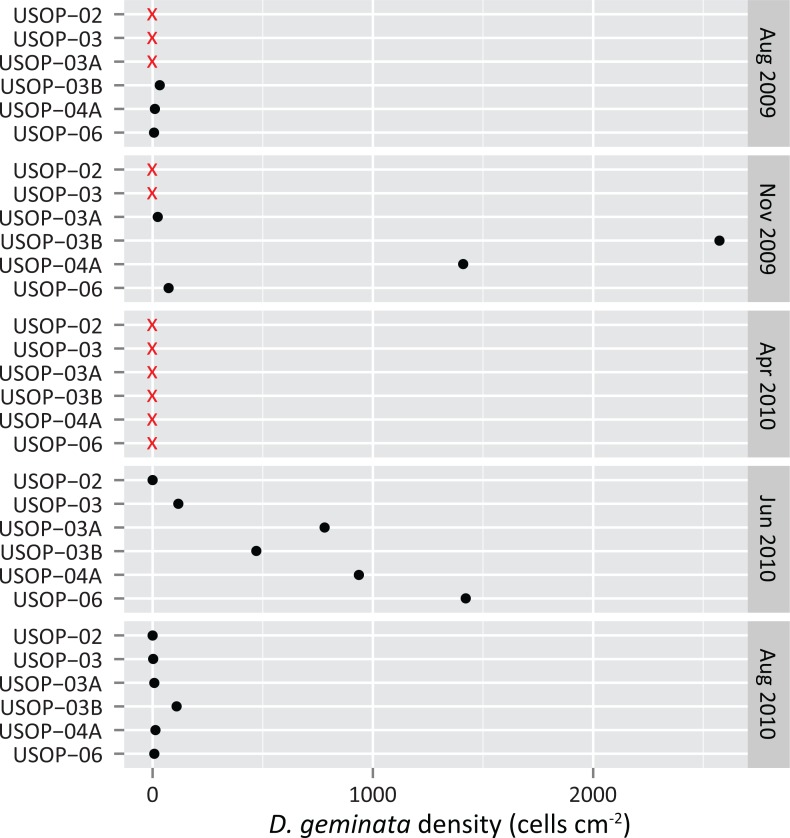
Cell density of *D*. *geminata* plotted by site and month showing a possible upstream range expansion. Black dots indicate cell density >0 and red “x”s indicate a non-detection.

Results of this investigation indicate that *D*. *geminata* may have expanded its range across parts of the watershed during the 12-month study. This seems likely because *D*. *geminata* was only identified in this highly monitored river system three months prior to our first (August 2009) survey. During this survey, *D*. *geminata* was only detected at main stem sites downstream of the Shandaken Portal (USOP-03B, USOP-04, USOP-04A, USOP-04B, USOP-05 and USOP-06) and at three tributary sites (STOC-00, STOC-01, and WODC-01) which all enter the Upper Esopus downstream of the Portal (Table 3). During the subsequent November 2009 survey, *D*. *geminata* was collected at the same three seasonal main stem sites, and at USOP-03A (Fig 5). This survey was the first to collect *D*. *geminata* upstream of the Shandaken Portal. *D*. *geminata* was not detected at any site during the April 2010 survey, but was found at all six of the seasonal main stem sites during June 2010, marking its first detection at USOP-02 and USOP-03. During the August 2010 survey, *D*. *geminata* was collected again at all six seasonal main stem sites (although the only detection at USOP-02 was a qualitative fourth replicate not included in this analysis), at additional downstream sites (USOP-04, USOP-04B, and USOP-05), and at three tributary sites (STOC-00, STOC-01, and BSNL-01). These findings suggest the diatom expanded its range on the main stem from a 17 km reach exclusively downstream of the Portal in August 2009 to an additional 2 km upstream of the Portal in November 2009, and to at least another 6 km upstream of the Portal by June 2010. *D*. *geminata* was not detected at the uppermost main stem site (USOP-00) or at six of the tributary sites during either of the comprehensive August surveys.

## Discussion

Though spatiotemporal variations of *D*. *geminata* in the Upper Esopus Creek watershed were significant and complex, blooms generally did not reach nuisance levels that would be expected to cause serious ecological effects. In streams from other regions with nuisance blooms, concentrations of AFDM and chl *a* often increased by a factor of 5–10 and exceeded guidelines for maximum desirable periphyton growth [2, 12]. For example, Kilroy [12] found that mean AFDM increased from 6.7 mg cm^-2^ to 33.2 mg cm^-2^ and mean chl *a* increased from 8.4 μg cm^-2^ to 45.3 μg cm^-2^ in stream reaches with *D*. *geminata* blooms compared to unaffected reaches of the Mararoa River, New Zealand. During our study in the Upper Esopus, densities of *D*. *geminata* and periphyton biomass were concurrently high during some periods (e.g., November 2009), but densities were also negligible during other surveys when periphyton biomass was high (e.g., April 2010). This suggests that the biomass of periphyton communities was not consistently dominated by *D*. *geminata* during the study. High densities of *D*. *geminata* only appeared to strongly affect standing crop during the November 2009 surveys at USOP-03B and USOP-04A. Additionally, *D*. *geminata* was only identified at four of the ten tributary sites: STOC-00 and STOC-01 during August 2009 and August 2010, WODC-01 during August 2009, and BNSL-01 during August 2010. Although it is possible that *D*. *geminata* has not been introduced to all of these tributaries, it is noteworthy that cell density never exceeded 13 cells cm^-2^ at any tributary site during August 2009 and was always below 1 cell cm^-2^ during August 2010 (Table 3). This observation may be similar to findings from a New Zealand study in which experimental introductions of *D*. *geminata* failed on spring-fed tributaries of larger rivers that supported *D*. *geminata* [42]. Even the highest densities of *D*. *geminata* at main stem sites of the Upper Esopus (2,574 cells cm^-2^ at USOP-03B and 1,410 cells cm^-2^ at USOP-04A during November 2009, and 1,422 cells cm^-2^ at USOP-06 during June 2010) were similar to or below those values reported in other North American studies [3, 43]. The lack of nuisance blooms in the Upper Esopus is significant because excessive periphyton growth can impact water quality, biodiversity, and the aesthetic and recreational value of a stream [44] which may affect local and regional economies [13].

The overall effect of *D*. *geminata* on diatom communities in the Upper Esopus appears limited. The relative abundance of *D*. *geminata* was consistently low and peaked at 1.3% of the entire diatom community. These findings are consistent with research on streams in the western United States where abundance of *D*. *geminata* never exceeded 3% of the entire diatom community [2]. Although density of *D*. *geminata* was positively correlated with total diatom density, results of the two-way ANOSIM indicate that the composition of diatom communities was not significantly affected by its presence or density. These results suggest that either *D*. *geminata* cell densities were not high enough to alter diatom communities or that cell density is a poor predictor of the impact of *D*. *geminata* on biota and benthic habitat. Since extracellular stalk material can comprise up to 90% of the *D*. *geminata* biomass [9], it is possible that a measure of *D*. *geminata* biomass or biovolume would have better identified changes to the diatom community.

The relatively low estimates of *D*. *geminata* cell density and standing crop during most surveys suggest that habitat or water quality in the Upper Esopus Creek watershed was not conducive to extensive bloom formation, at least during 2009 and 2010. It is well documented that bed-mobilizing high flows can scour algae from rock surfaces and effectively reset the successional process. Thus, the frequency of large floods can limit the growth and blooms of *D*. *geminata* [2, 18, 43, 45], in part because it is a late successional species [22]. Additionally, it has been shown that moderate and stable base flows are correlated with occurrence and abundance of *D*. *geminata* [46, 47], and the significant negative correlation between density of *D*. *geminata* and the discharge coefficient of variation in the present study further supports this finding. Prolonged blooms of *D*. *geminata* may be unusual within most tributaries and main stem reaches of the Upper Esopus upstream of the Portal because channel forming flows are common and summer base flows can be extremely low [48, 49]. In contrast, the extended periods of relatively stable base flows downstream of the Portal appear to favor proliferation of *D*. *geminata*. The only two periods (November 2009 and June 2010) when *D*. *geminata* reached high densities were preceded by relatively stable and moderate flows. Accordingly, no *D*. *geminata* cells were detected at any site during the April 2010 survey which followed a period of hydrologic instability and a peak discharge that exceeded 600 m^3^ s^-1^ at USOP-06. Other factors, however, had to be responsible for the low densities of *D*. *geminata* observed at most sites during the August 2009 and 2010 surveys because stream flows immediately preceding these surveys were comparatively stable.

**Fig 6:**
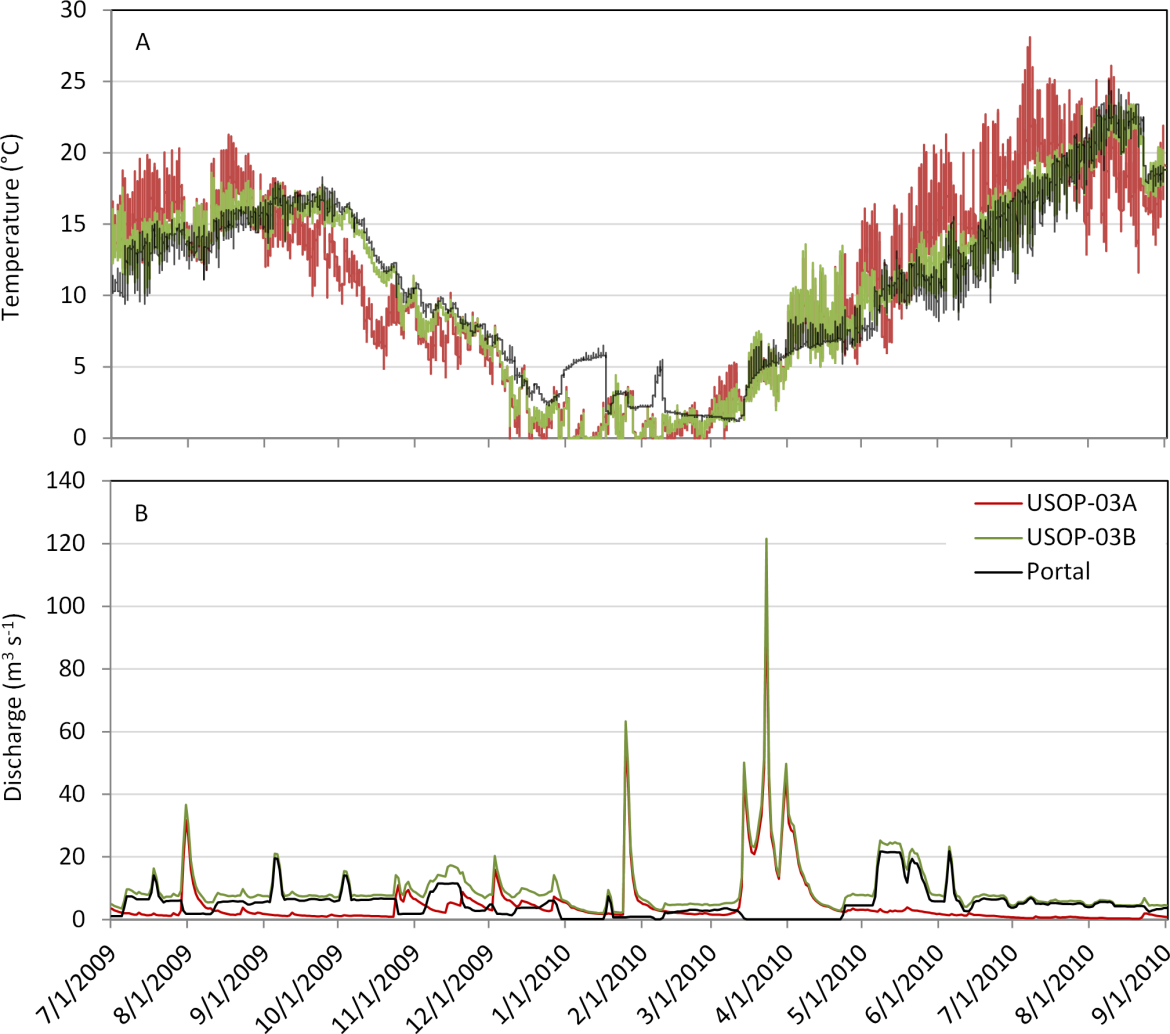
Continuous (15-minute) water temperature (A) and daily discharge (B) at USOP-03A (red), USOP-03B (green), and the Shandaken Portal (black) for the period 7/1/2009–9/1/2010.

High water temperatures may limit the growth and blooms of *D*. *geminata* and promote die back during mid to late summer in parts of the Upper Esopus Creek. Although the upper thermal tolerance of *D*. *geminata* appears variable [2], peak biomass of *D*. *geminata* has been linked to water temperatures that do not exceed 18°C [50] and *D*. *geminata* is more frequently found in locations where average summer air temperatures remain below 20°C [47]. Several laboratory studies using static tests confirmed that temperature was an important variable affecting the survival of *D*. *geminata* cells [51, 52]. Lagerstedt [51] found that cells were unable to survive more than 60 hours at 28°C and densities of viable cells gradually declined at 20°C. In the Upper Esopus, peak summer temperatures in the main stem consistently exceed 20°C. During 2010, water temperature at USOP-03A (and many other sites) exceeded 20°C for long periods of time and peaked at 28.1°C (Fig 6A). These temperatures far exceeded the preferred range of *D*. *geminata* and may be responsible for the low cell densities observed during the August 2010 survey.

The presence of *D*. *geminata* in the Upper Esopus is informative because suspended sediment concentrations are unusually high and blooms typically occur in clear oligotrophic streams with high light levels. Suspended sediment adversely affects lotic periphyton primarily through reduced light penetration [53]. Because the stalk length of *D*. *geminata* cells is positively correlated with light level [14], it follows that turbid waters could cause decreased stalk length and therefore less problematic blooms. It has also been demonstrated that abrasion caused by suspended sediment during elevated flows can scour and remove benthic algae [54]. However, the role of turbidity and suspended sediment in limiting the growth, density, and distribution of *D*. *geminata* is not well studied. In one of the few studies to identify sediments and turbidity as possible limiting factors, Kirkwood [3] found that moderate levels of turbidity and total suspended solids (means of 10.5 NTU and 9.35 mg L^-1^) may have restricted the growth of *D*. *geminata* in the Red Deer River (Alberta, Canada). In comparison, the median levels of turbidity and suspended sediment at 13 study sites in the Upper Esopus watershed from 10/1/2009 to 9/30/2010 ranged from 4.3 to 119.5 NTU and 3.0 to 136.0 mg L^-1^, respectively [55]. Each measure peaked at STOC-00 (where *D*. *geminata* was observed at low densities) and values were an order of magnitude higher than those in the Red Deer River. In general, it is apparent that waters across the Upper Esopus Creek watershed were similarly or more turbid than those of the Red Deer River and could, accordingly, have adversely affected the growth and density of *D*. *geminata*.

The supplemental flows from the Portal appeared to promote proliferation of *D*. *geminata* at downstream reaches of the Upper Esopus Creek during 2009 and 2010. The mean density of *D*. *geminata* downstream of the Portal was significantly greater than that of sites upstream of the Portal. In addition, mean density of *D*. *geminata* was highest at the site immediately downstream of the Portal (USOP-03B), and gradually declined at sites further downstream. Favorable thermal and hydrologic conditions produced by the Portal are likely responsible for these observations. Mean temperature from July 1 –August 31 was 16.0°C at USOP-03A and 14.8°C at USOP-03B in 2009 compared to 19.5°C and 19.3°C at these sites in 2010 (Fig 6A). Additionally, daily mean discharge at USOP-03A was usually below 2 m^3^ s^-1^ during both summers (lowest flow 0.24 m^3^ s^-1^, August 21, 2010), while the daily mean discharge at USOP-03B never dropped below 3.5 m^3^ s^-1^ during either summer (Fig 6B). Accordingly, the discharge coefficient of variation, which was negatively correlated with density of *D*. *geminata*, was smallest at USOP-03B when averaged across the five study periods (Table 2). Conditions at downstream reaches apparently become progressively less favorable as any beneficial effects of the Portal dissipate. These findings are not surprising because blooms of *D*. *geminata* have frequently been observed in reaches downstream of impoundments with regulated flow and thermal regimes [2, 9, 22, 23]. Stable base flows, like those present on the Upper Esopus immediately downstream of the Portal, are also considered beneficial for *D*. *geminata* [46, 47]. Therefore, it is likely that the moderated thermal and hydrologic conditions produced by supplemental flows from the Portal favor growth and sustained blooms of *D*. *geminata* in the downstream reaches of the Upper Esopus.

Although some recent studies indicate that blooms of *D*. *geminata* can alter invertebrate, algal, and fish communities, major ecosystem effects were not expected in the Upper Esopus Creek because of the low densities observed during most surveys. In other regions, the density of tolerant macroinvertebrates such as Chironomids and Oligochaetes increased, the number of Ephemeroptera, Plecoptera, and Trichoptera (EPT) taxa decreased, and overall community integrity declined in stream reaches impacted by blooms of *D*. *geminata* [10, 12, 56, 57]. Direct effects on fish assemblages have been more difficult to quantify and the deterioration of the brown trout (*Salmo trutta* L.) fishery in Rapid Creek, SD coincidental with the introduction of *D*. *geminata* in 2002 is one of the few studies to suggest fishery effects [9, 10, 57]. Although cell density across the Upper Esopus rarely exceeded 100 cells cm^-2^, densities >1,000 cells cm^-2^ were documented at sites downstream of the Portal during November 2009 and June 2010 and suggest that adverse impacts are possible. More recently (2012), Richardson et al [8] measured maximum *D*. *geminata* densities around 100,000 cells cm^-2^ in the Upper Esopus downstream of the Portal, and found that cell densities were negatively correlated with macroinvertebrate diversity, family richness, and EPT richness. Results from three companion studies on the Upper Esopus, however, did not identify any severe effects of *D*. *geminata* on resident fish and macroinvertebrate assemblages during 2009 through 2012. The mean New York State Biological Assessment Profile (BAP) score for the integrity of macroinvertebrate communities from eight samples downstream of the Portal from 2007 and 2008 (7.93) was nearly identical to the mean BAP score from 12 samples from 2009 and 2010 (7.94) after the appearance of *D*. *geminata* [58–60]. In addition, there were only minor differences in fish population or communities metrics at sites located upstream and downstream of Portal [61] (where densities of *D*. *geminata* were significantly different) and most were attributed to differences in habitat. Measures of physiological stress in brown trout were also lower or less evident at sites immediately downstream of the Portal (where *D*. *geminata* was most abundant) than at reaches upstream of the Portal [62].

Findings from several recent studies may further explain why dense blooms and elevated periphyton standing crop were not consistently observed in the Upper Esopus. The absence of dense sustained blooms and little indication of ecosystem impacts would intuitively suggest that habitat in this system is not optimal for *D*. *geminata*. Dense and problematic blooms, however, are apparently a response to phosphorous-limitation (soluble reactive phosphorus levels less than 2 mg m^-3^) in oligotrophic habitats [16, 17]. When phosphorous is not limiting, cell division rates increase for a short period of time, but stalk production decreases [15, 16] and *D*. *geminata* may be present in a non-nuisance capacity. Limited water samples collected from the Upper Esopus and tributaries during another companion study showed that orthophosphate ranged from roughly 6–11 mg m^-3^; which exceeds the 2 mg m^-3^ threshold for soluble reactive phosphorus identified in other studies. Additionally, macroinvertebrate samples from the Upper Esopus in 2007 and 2008 assessed using a Nutrient Biotic Index for phosphorous [63] suggest most main stem sites are mesotrophic [59]. Therefore, the relatively high levels of available phosphorus likely contribute, at least partly, to our conclusion that *D*. *geminata* did not dominate aquatic ecosystems in the Upper Esopus to the extent observed in other regions. A more comprehensive study comparing the behavior of *D*. *geminata* in the Upper Esopus to that from more oligotrophic streams of the same region would be needed to further evaluate and test this hypothesis.
