# Correction: *Didymosphenia geminata* in the Upper Esopus Creek: Current Status, Variability, and Controlling Factors

**DOI:** 10.1371/journal.pone.0134778

**Published:** 2015-08-05

**Authors:** The PLOS ONE Staff

Fig 1 is incorrect due to errors introduced during the typesetting process. The publisher apologizes for the error. Please see the corrected Fig 1 here.

**Fig 1:**
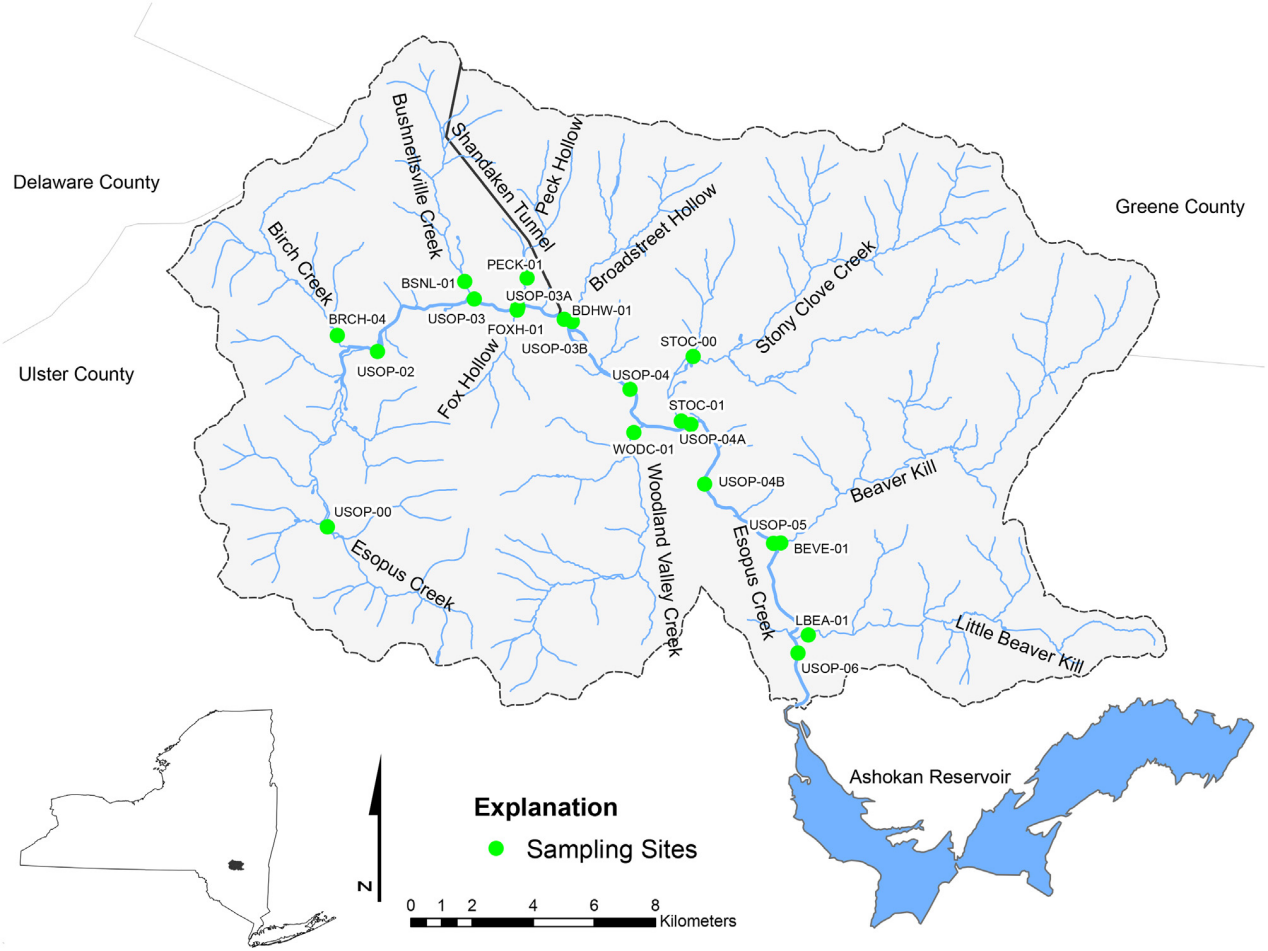
Locations of periphyton sampling sites in the Upper Esopus Creek and tributaries, 2009–2010.
